# Multisensory integration of dynamic emotional faces and voices: method for simultaneous EEG-fMRI measurements

**DOI:** 10.3389/fnhum.2013.00729

**Published:** 2013-11-14

**Authors:** Patrick D. Schelenz, Martin Klasen, Barbara Reese, Christina Regenbogen, Dhana Wolf, Yutaka Kato, Klaus Mathiak

**Affiliations:** ^1^Department of Psychiatry, Psychotherapy, and Psychosomatics, Medical School, Rheinisch-Westfaelische Technische Hochschule Aachen UniversityAachen, Germany; ^2^Jülich Aachen Research Alliance, Translational Brain MedicineAachen, Germany; ^3^Department of Neuropsychiatry, Keio University School of MedicineTokyo, Japan

**Keywords:** emotion, audiovisual integration, emotion integration, methods for EEG-fMRI, affective neuroscience, EEG-fMRI, perceptual processing

## Abstract

Combined EEG-fMRI analysis correlates time courses from single electrodes or independent EEG components with the hemodynamic response. Implementing information from only one electrode, however, may miss relevant information from complex electrophysiological networks. Component based analysis, in turn, depends on a priori knowledge of the signal topography. Complex designs such as studies on multisensory integration of emotions investigate subtle differences in distributed networks based on only a few trials per condition. Thus, they require a sensitive and comprehensive approach which does not rely on a-priori knowledge about the underlying neural processes. In this pilot study, feasibility and sensitivity of source localization-driven analysis for EEG-fMRI was tested using a multisensory integration paradigm. Dynamic audiovisual stimuli consisting of emotional talking faces and pseudowords with emotional prosody were rated in a delayed response task. The trials comprised affectively congruent and incongruent displays. In addition to event-locked EEG and fMRI analyses, induced oscillatory EEG responses at estimated cortical sources and in specific temporo-spectral windows were correlated with the corresponding BOLD responses. EEG analysis showed high data quality with less than 10% trial rejection. In an early time window, alpha oscillations were suppressed in bilateral occipital cortices and fMRI analysis confirmed high data quality with reliable activation in auditory, visual and frontal areas to the presentation of multisensory stimuli. In line with previous studies, we obtained reliable correlation patterns for event locked occipital alpha suppression and BOLD signal time course. Our results suggest a valid methodological approach to investigate complex stimuli using the present source localization driven method for EEG-fMRI. This novel procedure may help to investigate combined EEG-fMRI data from novel complex paradigms with high spatial and temporal resolution.

## INTRODUCTION

Combined EEG-fMRI investigates simultaneously neural activity at high spatial and temporal resolution (Mulert and Lemieux, 2010; Ullsperger and Debener, 2010). Early EEG-fMRI studies by Goldman et al. (2002) and Laufs et al. (2003) on the relationship between alpha power and BOLD signal investigated whether alpha power and BOLD signal are related. They correlated the time-series of occipital alpha during resting state with BOLD signal changes and reported an inverse correlation between occipital alpha power and BOLD signal in visual areas. Similar results in the visual cortex have been replicated in recent studies (Becker et al., 2011; Mayhew et al., 2013; Mo et al., 2013). In these studies, involvement of alpha oscillations in working memory (Scheeringa et al., 2009), linear superimposition in visual cortex (Becker et al., 2011) and default mode network (Mayhew et al., 2013; Mo et al., 2013) was investigated.

So far, two methods have been used to integrate alpha power in EEG-fMRI studies: correlation of single electrodes and of EEG components with the BOLD response. Single trial correlations of alpha power of single electrodes have been subject to several studies (Goldman et al., 2002; Laufs et al., 2003; Mo et al., 2013). They investigated neural correlations of occipital alpha oscillations in resting state with prior knowledge about the topography of the EEG signal. Other studies investigate neural correlates of ERPs and correlate single trial variation of one electrode with BOLD response. The EEG signal on the scalp can derive from several sources. Thus, this approach may impact the investigation of networks that are related to that scalp signal as the neural correlates of each electrophysiological source can not be identified.

Scheeringa et al. (2009) used a modified Sternberg paradigm to investigate the neural correlates of posterior alpha power increase during working memory maintenance. They conducted standard artifact reduction in EEG data and employed an independent component analysis (ICA). The component reflecting posterior alpha increase was chosen individually based on its topographical distribution. This was the first study to investigate single-trial coupling of alpha power and BOLD signal in a paradigm. However, subtle differences between different conditions may not be separable using ICA. Furthermore, if the topographical distribution of alpha power is unknown a priori, an ICA based approach to integrate EEG and fMRI data is not possible: Without a predefined topography, the component of interest cannot be identified. Therefore, we wanted to establish a way to identify neural correlates of electrophysiological oscillations during multisensory integration of emotions.

For complex stimuli – such as dynamic emotional multisensory stimuli, the exact topography of alpha oscillations – and particularly the subtle differences between emotional congruent (CON) and incongruent (INC) audio-visual stimuli is not known a priori. One solution for that is to use a source localization driven approach to identify those small distinctions and then combine EEG and fMRI data. In this pilot study, we will present a data-driven approach using EEG source localisation to analyse such complex multisensory information using EEG-fMRI. In a previous fMRI study on the integration of multisensory emotional stimuli, Klasen et al. (2011) compared CON and INC combinations of emotional dynamic faces and disyllabic pseudowords. They reported reduced workload on a fronto-parietal attention network for emotionally CON multisensory stimuli. In a magnetoencephalography (MEG) study, Chen et al. (2010) combined dynamic emotional faces with affective pseudowords. They reported increased alpha (8–13 Hz) power 200–400 ms after stimulus onset in frontal areas when comparing affectively CON multisensory to unisensory trials and concluded that multisensory integration of emotions occurred in higher order areas rather than in unisensory ones. Both studies report involvement of frontal areas during multisensory integration but lack either temporal (Klasen et al., 2011) or spatial resolution (Chen et al., 2010). Therefore, the exact spatio-temporal investigation of multisensory emotional integration with EEG-fMRI remains a challenging task: complex multisensory integration paradigms provide only a few trials per condition and the MR environment impacts the EEG signal-to-noise ratio (Laufs et al., 2003). Furthermore, the neural difference between emotionally CON and INC multisensory stimuli is expected to be rather small since early sensory processes are ruled out. Therefore, it needs to be established whether a source localization driven analysis of EEG and fMRI data suited to investigate neural processes during multisensory emotion integration on a trial-by-trial basis.

Based on the literature we used the following benchmarks for method validation:

(1) fMRI analysis will reveal robust activation of visual and auditory cortices after presentation of multisensory stimuli. Likewise, alpha power will be suppressed over occipital areas to show that alpha power was retained after EEG artifact rejection.
(2) An inverse relationship of alpha power in occipital areas and BOLD signal has been reliably reported in several EEG-fMRI studies. This relationship can be employed as a benchmark for a valid methodological approach. Thus, we hypothesize that stimulus induced alpha power suppression over occipital areas – as defined by source localization – will inversely correlate with BOLD response. This may confirm the technical feasibility of the here suggested data-driven approach for combining information of EEG and fMRI data.

In a next step, we provide evidence that this new approach may be suited to investigate the neural correlates of multisensory integration of emotions.

## MATERIALS AND METHODS

### SUBJECTS

Data was acquired from three male participants (P1: age: 21, P2: age: 24, P3: age: 22) for method demonstration. They reported normal vision, normal hearing, no contraindications against MR investigations, and no history of neurological or psychiatric illness. The participants were right-handed as assessed with the Edinburgh Handedness Inventory (Oldfield, 1971), german speaking and had normal intelligence according to multiple choice word test (MWT-B; Lehrl, 2005).

The experiment was designed according to the Code of Ethics of the World Medical Association (2008), and the study protocol was approved by the local ethics committee. Written informed consent was obtained and the participants were financially compensated for their participation.

### STIMULI

Audiovisual stimuli were dynamic angry, happy, and neutral virtual characters (avatars) combined with disyllabic pseudowords (angry, happy, or neutral prosody). Visual and auditory channels were combined in emotionally CON or INC fashion; the latter combined different auditory and visual emotions, e.g., a happy face with an angry pseudoword. Animated avatars were created with a 3D animation software package (Poser Pro, Smith Micro Software, CA, USA) and combined with the pseudowords using the incorporated Lip Synchronization Toolbox to assure lip and speech synchronization. The pseudowords followed German phonotactic rules and had no semantic content. Two female and two male avatars were associated with the voices of two male and two female speakers, with each avatar-voice combination being unique. Additionally, the avatars and the pseudowords were presented as visual-only and auditory-only stimuli, respectively. These stimuli have been validated and employed in a previous study (Klasen et al., 2011).

### TASK AND PROCEDURE

All stimuli were displayed using Presentation software (Neurobehavioral Systems, Inc, Albany, California, USA). A hybrid fMRI design of blocks for modality and events for emotions was used. The stimuli were grouped in 32 blocks (8 auditory, 8 visual and 16 audiovisual blocks), separated by a jittered pause of 19–21 s duration. Each block contained 12 trials resulting in a total of 384 stimuli (96 auditory, 96 visual and 192 audiovisual, balanced for emotion and gender). Audiovisual blocks contained randomly distributed CON and INC stimuli. Blocks and trials in each block were presented in pseudo-randomized order.

**FIGURE 1:**
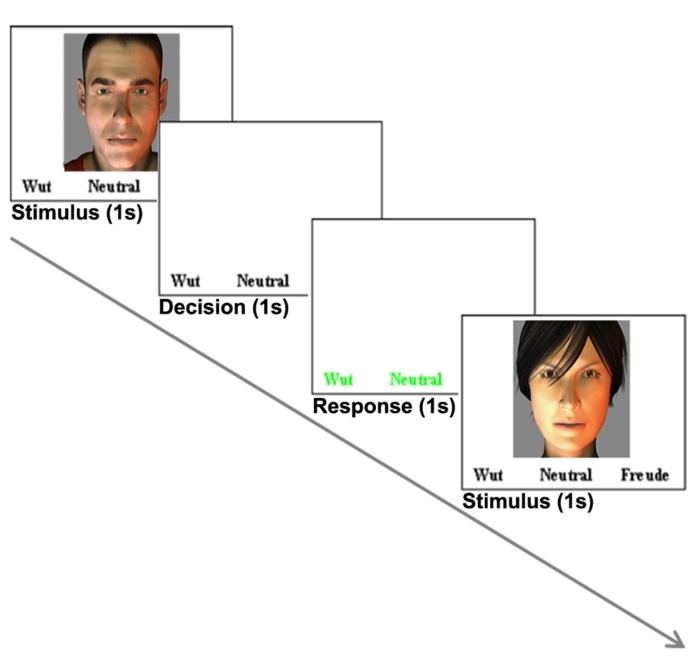
**Example of one trial.** Each trial (3–3.2 s) comprised a stimulus (1–522; 1.2 s), a decision phase (1 s) and a response phase (1 s). This example shows a visual trial. During trials with auditory stimulation only, the text was displayed.

Each trial started with a stimulus (1–1.2 s) followed by a decision phase (1 s) and a response phase (1 s), during which the participants had to rate the stimulus (delayed response design). The three different response options were displayed during stimulus and decision phase in a white color. The response phase was indicated by a change of colors to green (**Figure 1**) and the participants were instructed to rate the stimulus as a whole in the response phase as fast as possible. Responses were given by button pressing with index, middle and ring finger of the right hand.

### fMRI

Magnetic resonance imaging was conducted on a 3 Tesla Siemens Trio scanner (Siemens Medical, Erlangen, Germany). One run of echo-planar imaging (EPI) sequence acquired 34 transversal slices (TR = 2000 ms, TE = 28 ms, flip angle = 77°, voxel size = 3 × 3 mm with 64 × 64 matrix, 3 mm slice thickness, 0.75 mm gap). A radio frequency transmit-receive birdcage head coil allowed for simultaneous EEG recording. After the functional measurements, a high resolution, whole brain anatomical image was acquired with a 12-channel head coil (MPRAGE, T1-weighted, TE, 2.52 ms; TR, 1900 ms; flip angle, 9°; FOV, 256 × 256 mm; 1 mm isotropic voxels; 176 sagittal slices).

### fMRI PRE-PROCESSING

Image analysis was performed with *BrainVoyager QX 2.6* (Brain Innovation, Maastricht, the Netherlands). Pre-processing of functional MR images included slice scan time correction, 3D motion correction, spatial smoothing (4 mm FWHM), and high-pass filtering including linear trend removal. The first two images were discarded to avoid T1 saturation effects. Functional images were co-registered to 3D anatomical images and transformed into Talairach space. Trials without a response were omitted from further analysis. For auditory, visual, and CON audiovisual trials, only trials with correct responses were included. For INC audiovisual stimuli, all responded trials were included irrespective of correctness. All omitted and incorrect trials were modeled as a confound predictor in the GLM. Cluster threshold was determined with Monte-Carlo simulation (10000 iterations) as implemented in *BrainVoyager QX 2.6*.

### EEG ACQUISITION

Simultaneously with the fMRI acquisition, EEG was recorded from a 64-channel MR-compatible EEG-cap (Easycap GmbH, Herrsching-Breitbrunn, Germany) connected to a MR-compatible amplifier system (two BrainAmp MR plus 32-channel amplifiers, BrainProducts GmbH, Gilching, Germany). The EEG cap consisted of 64 Ag–AgCl electrodes (5 kΩ resistors), 63 of which covered the 10–20 system and an additional electrocardiogram (ECG) electrode placed below the left collar bone. Midline electrodes anterior and posterior to Fz served as the recording reference and ground channel, respectively. Prior to measurement, all channel positions were digitized using ELGuide V1.8 (Zebris Medical GmbH, Baden-Württemberg, Germany). Channel impedances were kept below 10 kΩ. To improve MR pulse artifact removal, a sync box (BrainProducts GmbH, Gilching, Germany) was used for optimal synchronization of EEG recording with the clock controlling MRI slice acquisition. EEG data were recorded in BrainVision Recorder software (v 1.05, BrainProducts GmbH, Gilching, Germany) at 5000 Hz sampling frequency (0.01–250 Hz analog band-pass filter) and analyzed in BrainVision Analyzer software (Version 2.02, BrainProducts, Gilching, Germany).

### EEG PRE-PROCESSING

**FIGURE 2:**
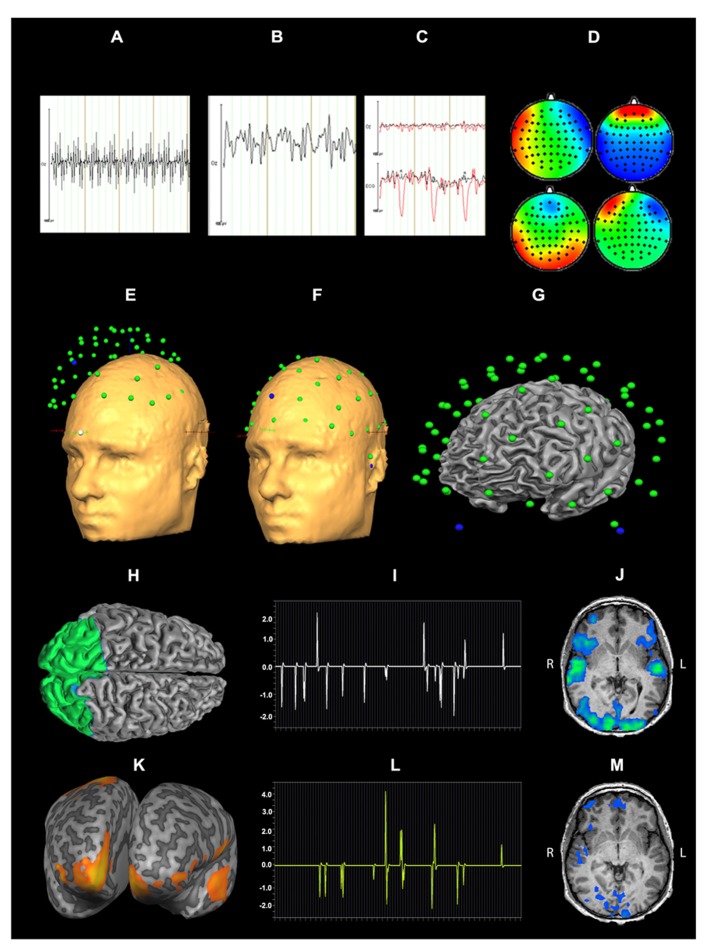
**Data processing for EEG-fMRI integration: EEG artifact removal included subtraction of MR pulse (A: before MR pulse subtraction, B: after MR pulse subtraction), cardioballistic artifact (C, red: before artifact subtraction, black: after artifact subtraction), eye blinks, head movement and residual BCG (D)**. 3D EEG channel coordinates were coregistered to Talairach space **(E,F)**. Successful co-registration of Talairach transformed EEG channel positions was confirmed by visual inspection **(G)**. Source localization of alpha power for audiovisual stimuli (CON + INC) revealed alpha power suppression in occipital areas **(H)**. This cluster was determined as a patch of interest to calculate alpha power time course: this time course was convolved with hemodynamic response function **(I)** and correlated with BOLD signal **(J)**. Contrast for (CON > INC) was estimated **(K)** and the alpha power time course in frontal region convolved with HRF **(L)** was used for correlation with BOLD signal time course **(M)**.

Pre-processing of EEG data included gradient artifact removal using a template subtraction algorithm (Allen et al., 2000). After gradient artifact removal (**Figures 2A,B**), the data were low-pass-filtered with a digital infinite impulse response filter (IIR, 70 Hz, 48 dB slope) and down-sampled to 500 Hz. Cardiac pulse correction was carried out based on an automatically detected pulse template in the ECG channel. Cardiac pulse markers were visually confirmed and the BCG artifact was subtracted (Allen et al., 1998; **Figure 2C**). Data sets were then down-sampled to 250 Hz and artifacts exceeding ± 300 μV were rejected. To remove artifacts due to eye movements, eye blinks and residual BCG artifacts, an ICA was conducted using 63 independent components. Components reflecting artifacts (**Figure 2D**) were visually identified and rejected based on topography and time course. All EEG channels re-referenced to average reference and pseudo-electrodes AFz and FCz were calculated using spherical interpolation resulting in a total of 65 channels. For EEG analysis, data were processed with BrainVision Analyzer software (Version 2.02, BrainProducts, Gilching, Germany). For EEG-fMRI integration, EEG data were exported to BrainVoyager QX 2.6.

### EEG ANALYSIS

Stimulus markers were imported based on Presentation timing log files (Neurobehavioral Systems, Inc, Albany, CA, USA). Trials without a response, errors, presentation uncertainty above 10 ms or amplitudes exceeding ± 125 μV were omitted from further analysis. Segmentation was based on stimulus onset (-2.000 to +1.000 ms) for auditory, visual, audiovisual CON and INC stimuli. Frequency decomposition was achieved by continuous wavelet transformation (complex morlet motherwavelet, *c* = 4.2) and baseline corrected (-1.500 to -500 ms). Further, segment’s average and standard deviation of alpha power was calculated. For representation of alpha power topography, wavelets with a center frequency of 10.5 Hz (borders: +/-2.5 Hz, wavelet length = 133 ms) were extracted.

### EEG-fMRI ANALYSIS

For EEG-fMRI coupling, the EMEG toolbox, implemented in *BrainVoyager QX 2.6*, was used. Talairach transformed anatomy was used for individual head surface and cortex mesh reconstruction. Individual 3D EEG channel coordinates were coregistered to head surface mesh for transformation to Talairach space (**Figures 2E,F**) and successful coregistration of EEG channel positions was visually confirmed (**Figure 2G**). Cortex meshes for both hemispheres were reconstructed using the outer gray matter boundary and the number of vertices was reduced to 2500 per hemisphere. Lead fields for each EEG channel were estimated assuming a four layer spherical head model (Berg and Scherg, 1994). A combination of three surface maps (in *x*, *y* and *z* direction) represented the channel specific lead field. To calculate the regularization term of the inverse solution, a 65 × 65 covariance matrix for -2000 to 0 ms (stimulus onset) was calculated which contained the spatial distribution of noise and spatial correlation of EEG channels. For estimation of an inverse solution to the EEG inverse problem (Hämäläinen and Ilmoniemi, 1994), we used the weighted-minimum norm solution with noise-based normalization as proposed by Dale et al. (2000). For a given SNR 5, the regularization parameter λ (Tikhonov and Arsenin, 1977) was estimated as 0.34 (accounting for 0.17% of the trace) to minimize noise amplification. Variation of the SNR between 1 and 10 did not yield relevant changes in the regularization.

### EEG-SOURCE ANALYSIS

Time series of alpha power (8–13 Hz) were calculated in *BrainVoyager QX2.6* for emotionally CON and INC multisensory stimuli using short time Fourier transformation (STFFT, Portoff, 1980) with the following settings: One time window consisted of 500 ms and was shifted for 100 ms resulting in 80% overlap between two neighboring windows The alpha power values were estimated from -2000 to 1000 ms after stimulus onset and baseline corrected from -2000 to 0 ms. Statistical maps of distributed EEG sources were estimated for affective multisensory stimuli. Separate Contrasts for audiovisual (CON + INC) trials over baseline and for CON over INC trials (CON > INC) were calculated: The first contrast tested preservation of alpha power after preprocessing and the second one evaluated a putative facilitation effect for CON multisensory information. The resulting clusters for (CON + INC) > baseline and the frontal cluster for (CON > INC; **Figures 2H,K**) served as a patch of interest (POI) for the correlation of alpha power and BOLD response in affective multisensory trials to provide evidence for a valid and sensitive methodological approach (**Figures 2H,I,J**).

### SINGLE TRIAL EEG-fMRI COUPLING

Single-trial induced alpha power at each event for 200–400 ms was calculated for the occipital and prefrontal cortex POI. Alpha power values were convolved with the hemodynamic response function and predicted the BOLD signal in a general linear model (GLM; **Figures 2I,L**). Induced power values of alpha oscillations considered the inverse solution of each POI incorporated the inverse solution and weighted the influence of all electrodes based on the inverse solution. Episodes without an event were set to zero. The definition of entire regions for correlations was based on results of the EEG source localization (see **Figures 2H,K**). Correlation maps for each occipital alpha power and prefrontal alpha power of CON and INC stimuli were estimated using first level statistics to identify neural sources that were related with the induced alpha in occipital and frontal areas (**Figures 2J** and **M**, respectively).

## RESULTS

### BEHAVIOR

On average, the participants correctly identified emotions in 70.8% of the prosodic trials and in 93.8% of the visual trials. Multisensory CON emotions were correctly classified in 92.9%. In the INC condition, the participants decided in 51.0% of the stimuli according to the facial expression and in 28.4% according to the emotional prosody and in 22.6% for neither facial nor auditory emotion.

### fMRI

**FIGURE 3:**
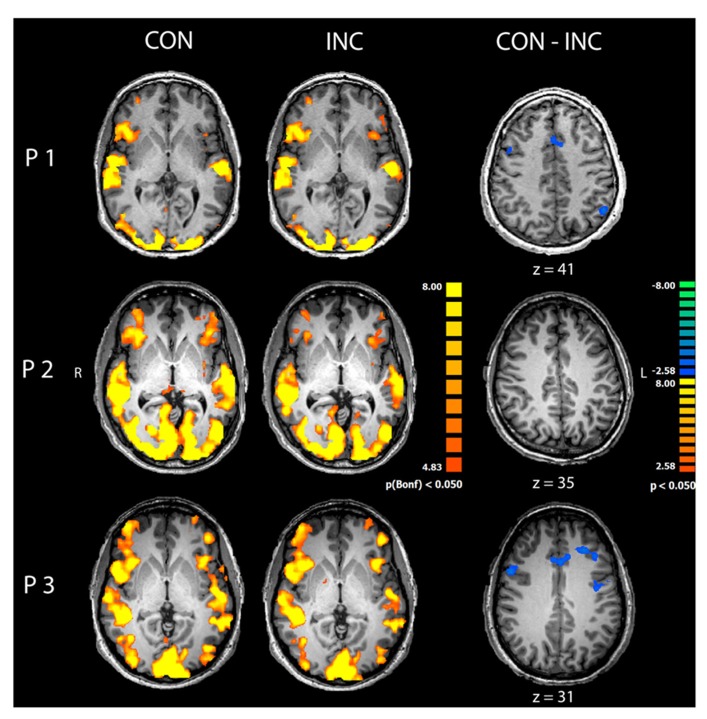
**Comparison of congruent and incongruent trials.** Congruent (CON) and incongruent (INC) trials elicited highly significant widespread activations in visual and auditory cortices as well as motor and medial frontal areas in participants P1–P3 (*p *< 0.05, Bonferroni corrected). Incongruent compared to congruent trials induced significantly higher activity in mediofrontal and dorsolateral regions in participants P1 and P3 (CON-INC, *p* < 0.05, cluster-threshold corrected).

Congruent and INC trials compared to baseline yielded significantly increased activity in bilateral visual, auditory, frontal and motor areas (*p* < 0.05, Bonferroni corrected; see **Figure 3**, CON and INC). The comparison of CON and INC trial revealed significantly higher activation for INC trials in ACC and dorsolateral-prefrontal cortex in two out of three participants (**Figure 3**, CON-INC; *p* < 0.05; cluster-threshold corrected).

### EEG

**FIGURE 4:**
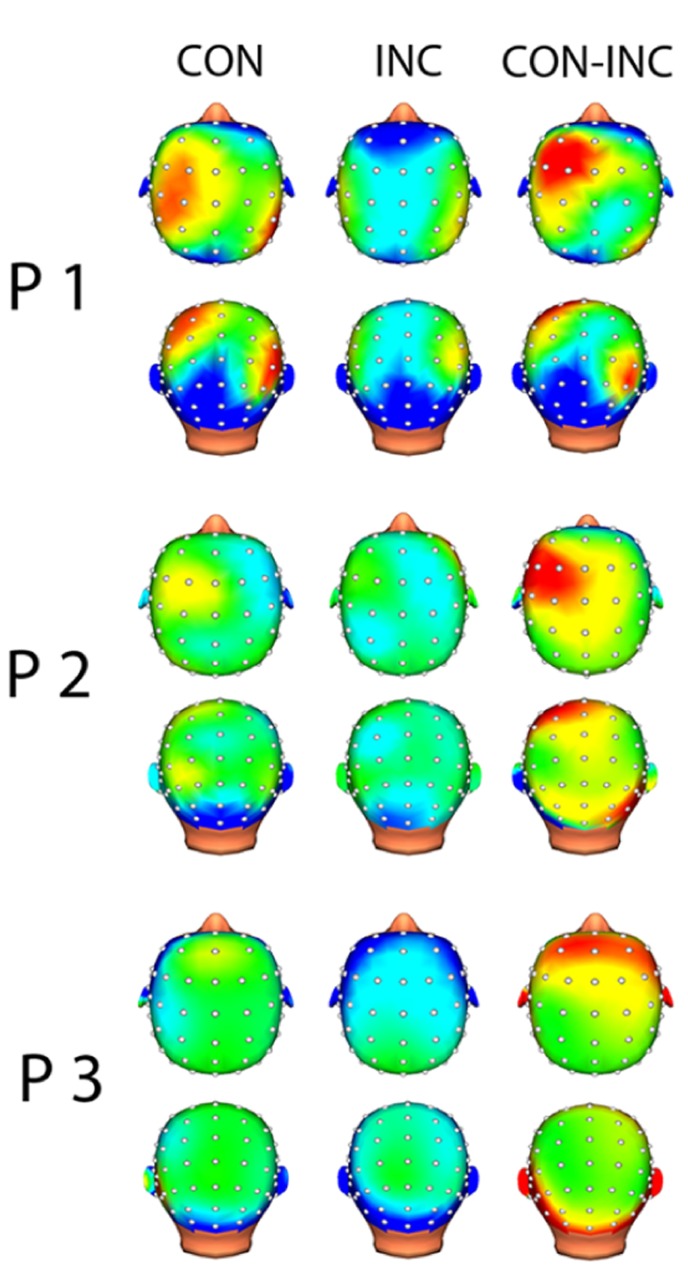
**Topographic map of induced alpha power 200–400 ms after multisensory stimulus onset.** Increased alpha power is displayed in warm colors, reduced alpha power in cold colors. Topographical distribution of alpha oscillation showed frontal increased alpha power for CON and slightly reduced alpha power in central electrodes for INC. Difference between both multisensory stimuli indicated higher induced alpha power for CON.

In each channel, less than 10% of the data points were excluded due to artifact rejection in all participants. EEG analysis suggested alpha power suppression over occipital areas for both CON and INC trials (**Figure 4**, CON-INC) and elevated induced alpha oscillations for CON stimuli in frontal areas during 200–400 ms after stimulus onset (**Figure 4**, CON). In contrast to CON stimuli, Incongruency of emotions induced alpha power suppression in a small cluster at the Fz electrode (**Figure 4**, INC). The difference between induced alpha power of CON and INC stimuli 200–400 ms after stimulus onset yielded a left lateralized frontal cluster (**Figure 4**, CON-INC).

#### EEG source analysis

**FIGURE 5:**
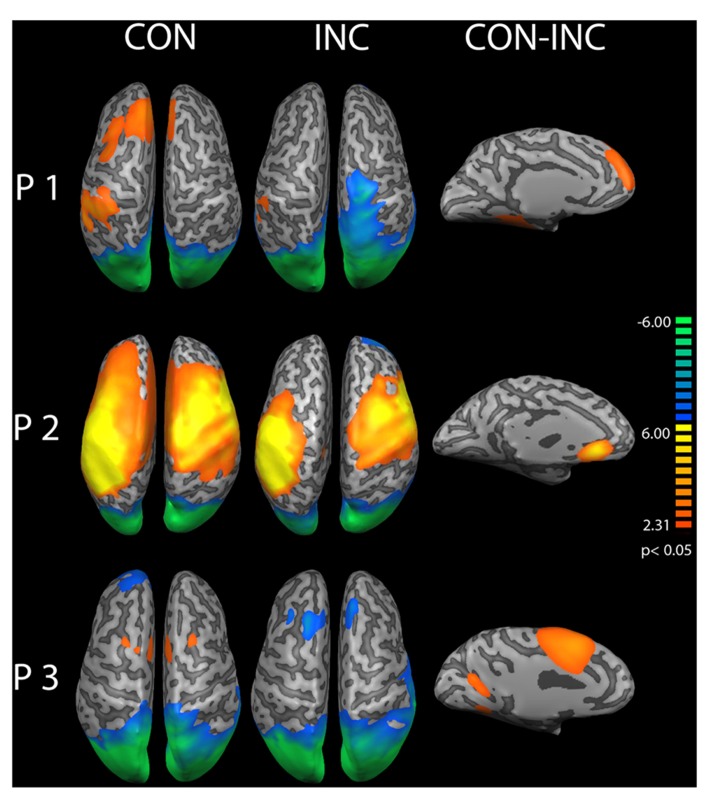
**Source analysis of induced alpha power during 200–400 ms after stimulus onset.** Increased alpha power over baseline is displayed in warm colors, reduced alpha power in cold colors for congruent (CON) and incongruent (INC) stimuli (*p* < 0.05). In partici-pants P1–P3, stimulus presentation suppressed alpha power over the occipital lobe. Congruent trials elicited higher alpha power in mediofrontal regions. A comparison of congruent over incongruent stimuli showed significant (CON, *p* < 0.05) higher induced alpha power for congruent trials in frontal areas in the left hemisphere (CON-INC, *p* < 0.05).

A cortical constrained minimum-norm-weighted inverse solution estimated the sources (Grech et al., 2008). Alpha power was suppressed over the occipital cortex (OC) about 200–400 ms after onset of audiovisual stimuli (**Figure 5** CON and INC). The topography confirmed preservation of the alpha oscillations after EEG preprocessing whilst successful artifact removal, therefore allowing the investigation of differences between induced alpha power of CON and INC stimuli. For CON stimuli, higher alpha power extended bilaterally to fronto-medial areas (**Figure 5**, CON) whereas INC stimuli induced alpha power suppression at the frontal cortex in participants P2 and P3 (**Figure 5**, INC).

A contrast for (CON > INC) was estimated to test whether previous results by Chen et al. (2010) could be replicated. It revealed significantly elevated induced alpha power 200–400 ms after stimulus onset for CON stimuli in left prefrontal cortex (**Figure 5**, CON-INC; *p* < 0.05). No further difference for (CON > INC) was observed during later time windows (600–800 ms and 800–1000 ms). As a next step, the time series of single trial induced alpha power for the OC and frontal areas were individually correlated with whole brain BOLD signal to identify neural networks supporting the event-related changes of alpha oscillations.

### EEG-INFORMED fMRI

#### Correlation analysis of induced alpha power in OC

**FIGURE 6:**
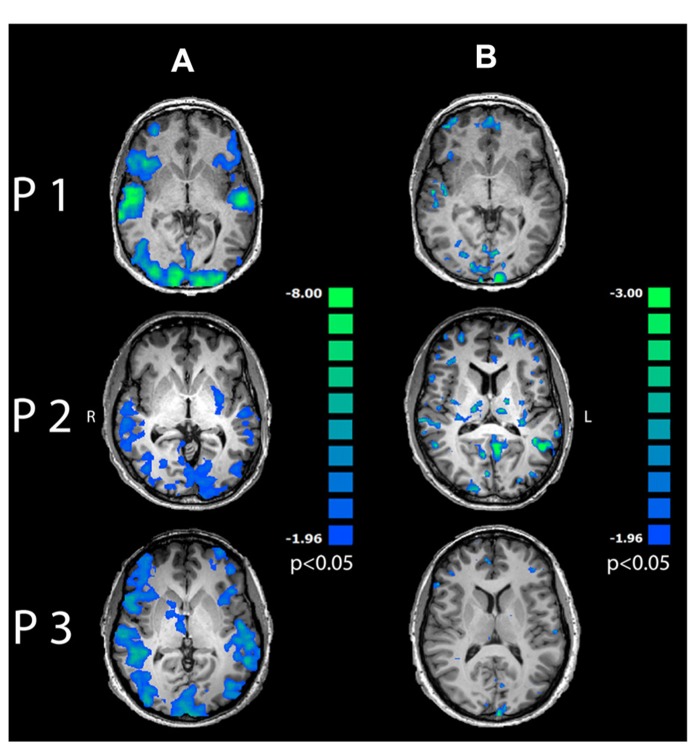
**Regression maps between induced alpha power during 200–400 ms after stimulus onset and BOLD response for multisensory stimuli.** Negative correlations are displayed in cold colors. **(A)** Induced alpha power over occipital lobe correlated significantly with BOLD response to multisensory emotions in visual, auditory and frontal areas (*p *< 0.05, cluster-threshold corrected). **(B)** Induced alpha power in PFC correlated negatively with BOLD response in mediofrontal, dorsolateral and occipital areas (*p* < 0.05, uncorrected).

Single-trial variability of induced alpha power to multisensory stimuli in the OC correlated negatively with BOLD response in a wide-spread network encompassing visual and auditory cortices, dorsolateral and prefrontal areas as well as bilateral insula (**Figure 6A**; *p* < 0.05, cluster-threshold corrected).

Further correlation analysis between time series of induced alpha power during 200–400 ms after presentation onset in the PFC and BOLD response revealed exclusively inverse correlations in mediofrontal, dorsolateral, prefrontal and visual areas (**Figure 6B** and Table 1, *p* < 0.05, uncorrected) to emotionally INC stimuli.

**Table 1:** Decisions for uni- and multisensory emotional trials.

	Auditory trials (%)	Visual trials (%)	Congruent trials (%)	Decision for face (%)	Decision for voice (%)
P1	69.3	92.0	96.1	39.6	40.6
P2	75.4	96.2	93.8	57.3	23.6
P3	72.2	95.5	89.8	62.3	21.0

## DISCUSSION

The aim of this pilot study was to investigate the technical feasibility of a source analysis-driven approach to integrate the high spatial accuracy of fMRI and high temporal resolution of EEG using simultaneous EEG-fMRI in a fast, event-related design. We confirmed EEG and fMRI data quality separately by reproducing established response patterns in all participants. During the presentation of multisensory affective trials the fMRI data analysis revealed strong activation in a distributed network encompassing visual, auditory cortex and insula in accordance with previous studies (Ethofer et al., 2006; Klasen et al., 2011) and confirms reliable fMRI data registration despite the simultaneous EEG recording. In a similar vein, it is known that MRI environment is detrimental on EEG data quality (Mullinger and Bowtell, 2011) before various artifact removal steps are being applied (mainly correction of HF scanner artifact, correction of cardioballistic signal and eye blinks). The low amount of epoch rejection for multisensory stimuli suggested a successful artifact removal. However, there remains a speculative notion to this conclusion since we did not have any outside scanner data to directly compare data quality. A source analysis was conducted of induced alpha oscillations for multisensory trials to test for the preservation of alpha oscillations after EEG processing. Topography of induced alpha power in occipital areas during 200–400 ms after multisensory stimulus onset confirmed preservation of alpha oscillations and indicated increased cortical activation due to complex sensory input. Shagass (1972) assumed that alpha oscillations are an indirect measurement of cortical activity which has been supported by recent studies (Klimesch, 1999; Nunez et al., 2001; Jokisch and Jensen, 2007; Palva and Palva, 2007). Therefore, reduced alpha power over occipital areas is likely to indicate effective signal processing at sensory cortices (Mathiak et al., 2011). The first EEG-fMRI studies on the relationship of alpha power and BOLD response (Goldman et al., 2002; Laufs et al., 2003) reported an inverse correlation of occipital alpha oscillations and BOLD signal in line with previous hypothesis about the function of the alpha oscillations as an idling or suppression rhythm (Mazaheri and Jensen, 2010). This negative correlation has also been used as a benchmark for a valid methodological approach in recent EEG-fMRI studies (Scheeringa et al., 2009; Mayhew et al., 2013, Mo et al., 2013). In this study, we replicated a reasonable correlation pattern between occipital alpha power suppression and BOLD signal, thus confirming the validity of our source localization driven method.

But for which investigations can the presented procedure be used? Current studies apply two methods: correlation of single electrode time course (e.g., Oz) or individual EEG components with BOLD response. In a sophisticated study, Scheeringa et al. (2009) investigated working memory networks related to alpha power using EEG-fMRI. The authors applied an ICA approach to extract a components reflecting alpha power time course based on its topography. This method enhances SNR due to exclusion of noise which remains in the other components. But this analysis depends critically on a priori knowledge about the topography of the EEG signal which makes this approach unfeasible for investigating new paradigms. The selection of single electrodes is frequently employed in ERP studies to calculate correlations with the BOLD signal (e.g., Eichele et al., 2005; Dubois et al., 2012). In contrast to this more common ERP analysis, the present analysis incorporates multichannel information. Hence, different components of a network as identified by source analysis and their neural correlates may be investigated using EEG-fMRI.

Studies investigating multisensory integration usually identify neural networks and deal with only subtle differences between conditions where the topography of the EEG signal is unknown a priori. Therefore the classical procedures are not suitable and a source localization-driven, sensitive method is necessary to investigate the time course of multisensory integration of emotions with high spatial and temporal resolution using EEG-fMRI. In an explorative analysis, we further tested the feasibility of this method to investigate multisensory integration of emotions. Chen et al. (2010) reported in an MEG a facilitation effect for multisensory emotional stimuli 200–400 ms post stimulus presentation. Therefore we verified our method further in the context of multisensory integration. Our behavioral data are generally in line with previous studies reporting high visual and CON multisensory recognition rates and slightly lower ones for the auditory-only stimuli (Vroomen et al., 2001; Campanella and Belin, 2007; Klasen et al., 2011). This confirmed that affective pseudowords, faces and their combination were identified correctly on a behavioral level. The missing facilitation effect between visual-only and CON multisensory stimuli may be attributed to a ceiling effect since recognition rates for both were very high (>90%). Using first level statistics, a significant facilitation effect for CON stimuli was found in PFC for early (200–400 ms) but not for late (600–800 ms) induced alpha oscillations during early perceptual processing. This power difference disappeared after 600 ms, reproducing a previous MEG study (Chen et al., 2010). They reported a facilitation effect of affective audiovisual processing over both auditory and visual stimuli in a similar time window only (200–450 ms). The here presented reproduction based on only few stimuli in a single subject further corroborates the efficiency of the presented EEG analysis. We suggest that increased induced alpha power for CON stimuli may reflect reduced cognitive demand by stimulus disambiguation (Stein and Meredith, 1993; Ernst and Bülthoff, 2004) and perceptual processing of affective multisensory stimuli during 200–400 ms after stimulus onset. Essential information on the role of various brain structures in multisensory emotion integration comes from the trial-wise correlations of induced oscillations and BOLD signal, which constitutes the major benefit of simultaneous EEG-fMRI measurements. Combined EEG-fMRI indicated that the facilitation effect of early induced alpha power for emotional CON multisensory information is not constrained to frontal areas. Further contributions may origin in a fronto-medial and -lateral evaluation network which is known to be involved in processing of multisensory stimuli (for reviews, see Calvert and Thesen, 2004 and Klasen et al., 2012). The reproducible activation in the fronto-medial and -lateral network may reflect cognitive feature evaluation in early processing whereas this may not be necessary for CON stimuli as auditory und visual input confers redundant information.

## CONCLUSION

With our study we provided a novel method to investigate the temporal course of affective multisensory integration. To our knowledge, this is the first study so far that investigated the feasibility of a source localization driven approach of induced alpha power of affective multisensory processing with BOLD response in a fast event related design employing simultaneous EEG-fMRI. We demonstrated that the analysis of combined simultaneous EEG-fMRI recording provided valid and informative EEG, fMRI, as well as EEG-informed fMRI results.

Thus, the results support the technical feasibility of this novel approach and may help to disentangle the neural correlates of perceptual and decisional processing during multisensory integration of affective information.

## LIMITATIONS

This method relies – in contrast to previous ones – on the inverse solution of EEG data. The source localization using this method can deviate substantially from subject to subject. Therefore a high data quality and strict EEG artifact rejection is necessary to employ this method. Furthermore, we did not compare EEG scalp inside and outside the MR environment and interpretation about the topographies remains speculative. But we replicated known response patterns for alpha power after visual stimulus presentation and even confirmed the topography of induced alpha power to multisensory stimuli (Chen et al., 2010). Conceivably, the presented source localization driven method can specify small signal differences in empirical EEG-fMRI studies.

This pilot study included three subjects only. Although the results of EEG-fMRI combination yielded significance, a generalization of these exploratory findings is not possible so far.

## AUTHOR CONTRIBUTIONS

Patrick D. Schelenz and Martin Klasen designed the paradigm. Patrick D. Schelenz, Barbara Reese, Martin Klasen and Dhana Wolf acquired data. Patrick D. Schelenz, Yutaka Kato, Christina Regenbogen and Klaus Mathiak analyzed the data. Patrick D. Schelenz, Barbara Reese, Christina Regenbogen, Dhana Wolf, Yutaka Kato and Klaus Mathiak wrote the paper.

## Conflict of Interest Statement

The authors declare that the research was conducted in the absence of any commercial or financial relationships that could be construed as a potential conflict of interest.
