# Corrigendum: Multisensory integration of dynamic emotional faces and voices: method for simultaneous EEG-fMRI measurements

**DOI:** 10.3389/fnhum.2016.00624

**Published:** 2016-12-01

**Authors:** Patrick D. Schelenz, Martin Klasen, Barbara Reese, Christina Regenbogen, Dhana Wolf, Yutaka Kato, Klaus Mathiak

**Affiliations:** ^1^Department of Psychiatry, Psychotherapy, and Psychosomatics, Medical School, Rheinisch-Westfälische Technische Hochschule Aachen UniversityAachen, Germany; ^2^Jülich Aachen Research Alliance, Translational Brain MedicineAachen, Germany; ^3^Department of Neuropsychiatry, Keio University School of MedicineTokyo, Japan; ^4^Institute of Neuroscience and Medicine, Forschungszentrum Jülich GmbHJülich, Germany

**Keywords:** emotion, audiovisual integration, emotion integration, methods for EEG-fMRI, affective neuroscience, EEG-fMRI, perceptual processing

Reason for Corrigendum:

In the original article, a term “wrote the paper” appeared in the Author Contributions. This was unintentionally vague and did not differentiate between writing and correcting the manuscript. The authors apologize for the lack of clarity. This error does not change the scientific conclusions of the article in any way.

The corrected Author Contributions are as follows:

## Author contributions

PS and MK designed the paradigm. PS collected the data and BR, MK, and DW helped acquiring the data. PS analyzed the data. YK, CR, and KM supported data analysis and interpretation. PS wrote and all co-authors corrected the manuscript. All authors gave final approval of the version to be published.

### Conflict of interest statement

The authors declare that the research was conducted in the absence of any commercial or financial relationships that could be construed as a potential conflict of interest.

